# 

**Published:** 1852-07

**Authors:** 


### THE WEST.

JAMES TAYLOR M. D., D. D. S., EDITOR.

**PUBLISHED QUARTERLY;**

**BY ORDER OF THE MISSISSIPPI VALLEY ASSOCIATION OF DENTAL SURGEONS.**

CINCINNATI:
PRINTED BY JOHN D. THORPE,
NO. 74, WEST FOURTH STREET.
1852.

**TERMS---$2,00 PER ANNUM, PAYABLE IN ADVANCE.**

